# Supplementary material for: Minimization of Disorder as a Key Design Principle for Natural Sizes of Light Harvesting 2 Complexes
Source: J Phys Chem Lett. 2026 Jun 9;17(25):7028–38. doi: 10.1021/acs.jpclett.6c00794 (PMC13312445; doi:10.1021/acs.jpclett.6c00794)
Supplement: Supplementary file 1 [file jz6c00794_si_001.pdf]

Supporting Information: Minimization of disorder as a key design principle for natural sizes of light harvesting 2 complexes

Kwang Hyun Cho,[†] Seogjoo J. Jang,[*,‡,¶,†] and Young Min Rhee[*,§]

†*Korea Institute for Advanced Study, Seoul 02455, South Korea*

‡*Department of Chemistry and Biochemistry, Queens College, City University of New York, Queens, New York 11367, United States*

¶*Ph.D. Programs in Chemistry and Physics, Graduate Center of the City University of New York, New York, New York 10016, United States*

§*Department of Chemistry, Korea Advanced Institute of Science and Technology (KAIST), Daejeon 34141, South Korea*

E-mail: seogjoo.jang@qc.cuny.edu; ymrhee@kaist.ac.kr

Supporting Text

Quantum chemistry calculation

Throughout this work, quantum chemistry calculations were performed using density functional theory (DFT) method with the B3LYP functional, which has been widely adopted as a computationally efficient and numerically stable functionals with minimal parameters. We confirmed that B3LYP provides reliable results consistent with more sophisticated approaches for our specific systems, such as range-separated functionals[S1] or dispersion corrections[S2] (Figure S4). Besides, our IM part does not include π stacking nor protein-ligand interaction. Thus, inclusion of dispersion interaction at the DFT level was not deemed necessary. In fact, such interactions are actually accounted for in our IM/MM model with reasonable accuracy through the IM–MM interaction terms. Potential systematic errors associated with these functionals have been discussed and benchmarked in the literature.[S3]

Initialization and Preparation of the Simulation

The crystal structure of the LH2 complex (PDB ID: 2FKW) was adopted as our starting point, which naturally exhibits 9-fold symmetry. From the crystal structure, a single-unit monomer was extracted from the 9-fold symmetric complex. The monomers were then cylindrically arranged to construct a LH2 complex with any desired n-fold symmetry. The constructed complex was then solvated with TIP3P water model,[S4] accompanied by charge-neutralizing chloride ions. The entire solvated complex was placed at the center of a cubic simulation box with a side length of 10 nm. Next, we performed QM/MM energy minimization to optimize the structure using combination of GROMACS package[S5] with Q-Chem 5.0.[S6] During the optimization process, the π conjugation system of each BChls was treated as the QM region, using density functional theory (DFT) with the 6-31G(d,p) basis set and the B3LYP functional, and the rest of the pigment was modeled to closely replicate the Hessian matrix obtained from quantum chemical calculations.[S7,S8] The remaining part of the

system was described using the CHARMM27 force field parameters.[S9]

Summary of the IM method

In this section, we provide a brief overview of the PES-IM approach and relevant notations for completeness of our presentation. This will also provide a clear motivation for employing a pre-calculated dataset to approximate QM/MM calculations. Here, we denote the Cartesian coordinates as $\mathbf{X}$ and the internal coordinates as $\mathbf{Z}$. The data point comprising the IM PES for each index i consists of coordinate $\mathbf{X}_i$ and relevant properties such as energy E_i, gradient $\mathbf{g}_i$, and Hessian $\mathbf{h}_i$. To obtain the potential energy at a pigment geometry $\mathbf{Z}$, the energy is primitively approximated from each data point in the dataset using a single-point Taylor expansion:

$$V_i(\mathbf{Z}) = E_i + \mathbf{D}_i^{\mathrm{T}} \cdot \mathbf{g}_i + \frac{1}{2}\mathbf{D}_i^{\mathrm{T}} \cdot \mathbf{h}_i \cdot \mathbf{D}_i \tag{1}$$

where $\mathbf{D}_i = \mathbf{Z} - \mathbf{Z}_i$ represents the difference between the target geometry and the data point geometry $\mathbf{Z}_i$. The final potential energy is obtained as a weighted sum of the Taylor expansion from all data points:

$$V(\mathbf{X}) = \sum_i w_i(\mathbf{X}) V_i(\mathbf{Z}) \tag{2}$$

where $w_i(\mathbf{X})$ is a normalized weight based on a modified Shepard weighting function.[S11] For LH2, the π conjugation system of every BChl was treated as the IM region. A detailed explanation, particularly its integration with MM methods for IM/MM simulation can be found in the literature.[S7]

The IM approach requires a well-designed and robust sampling scheme for collecting its data points to ensure the accuracy and reliability of the PES. Conventionally, a primitive database is constructed with minimally available data points, and the database is iteratively

improved by adding more data points after performing preliminary simulations and sampling key configurations, often referred to as "GROW scheme".

Inter-LH2 exciton transfer dynamics

With a generalized master equation for modular exciton density (GME-MED), line shape functions which describe the influence of the dynamic disorder are computed as defined below:

$$\lambda_n = \int_0^\infty d\omega \frac{J_n(\omega)}{\omega} \tag{3}$$

$$G_{n,i}(t) = \frac{1}{\hbar}\int_0^\infty d\omega \frac{J_n(\omega)}{\omega^2}\sin(\omega t) \tag{4}$$

$$G_{n,r}(t) = \frac{1}{\hbar}\int_0^\infty d\omega \frac{J_n(\omega)}{\omega^2}\coth\left(\frac{\hbar\omega}{2k_B T}\right)(1-\cos(\omega t)) \tag{5}$$

where $J_n(\omega)$ denotes the spectral density of harmonic contribution for each type of BChl, as presented in the main text (Figure 4). Figure S5 shows the corresponding line shape functions for the B850 BChls of each symmetry.

The GME-MED method employs an approximation neglecting off-diagonal components in the exciton basis. Toward this, we introduce a transformation matrix between site basis of kth LH2, $|n_k\rangle$, to the exciton basis $|\phi_{p_k}\rangle$, $U_{n_k,p_k} = \langle n_k | \phi_{p_k}\rangle$. We also introduce reorganization energy and line shape functions in an exciton basis.

$$\lambda_{p_k} = \left(\sum_{n_k} |U_{n_k,p_k}|^4 \lambda_{s_n}\right) \tag{6}$$

$$G_{p_k,i}(t) = \left(\sum_{n_k} |U_{n_k,p_k}|^4 G_{n,i}(t)\right) \tag{7}$$

$$G_{p_k,r}(t) = \left(\sum_{n_k} |U_{n_k,p_k}|^4 G_{n,r}(t)\right) \tag{8}$$

Then, the energy transfer rate can be approximated as

$$W_{1\to 2}(t) = \frac{2}{\hbar^2}\mathrm{Re}\sum_{p_1}\sum_{p_2}\frac{e^{-\tilde{\epsilon}_{p_1}/k_BT}}{\left(\sum_{p_1'} e^{-\tilde{\epsilon}_{p_1'}/k_BT}\right)}\left|\tilde{V}_{p_1p_2}\right|^2$$
$$\times \int_0^t d\tau e^{-G_{p_2,r}(\tau)-iG_{p_2,i}(\tau)-i\tilde{\epsilon}_{p_2}\tau/\hbar}$$
$$\times \int_0^t d\tau e^{-G_{p_1,r}(\tau)-iG_{p_1,i}(\tau)+i\tilde{\epsilon}_{p_1'}\tau/\hbar} \quad (9)$$

with $\tilde{\epsilon}_{p_k} = \epsilon_{p_k} - \lambda_{p_k}$ and $\tilde{V}_{p_1p_2} = \sum_{n,m} U_{n_2,p_2}V_{n_1,m_2}U^*_{m_1,p_1}$.

At the initial condition, the exciton is in thermal equilibrium in the first LH2. The exciton dynamics between two B850 rings follows

$$\frac{\partial}{\partial t}p_1(t) = W_{2\to 1}(t)p_2(t) - W_{1\to 2}(t)p_1(t) \quad (10)$$

With an exciton partition function of the kth LH2, $Z_k = \sum_{p_k} e^{-\tilde{\epsilon}_{p_k}/k_BT}$, an effective forward rate is defined as

$$k_f = \frac{Z_2}{Z_1+Z_2}\frac{1}{\tau_1} \quad (11)$$

where a transfer time τ_1 is the shortest time satisfying

$$\ln\left(p_1(\tau_1) - \frac{Z_1}{Z_2}p_2(\tau_1)\right) = -1 \quad (12)$$

Table S1: Partial charges of BChl in the ground state. Atomic indices follow the reference.[S10]

	6-fold			9-fold			12-fold		
	α	β	γ	α	β	γ	α	β	γ
1	1.47	1.47	1.52	1.47	1.63	1.41	1.45	1.60	1.55
2	−0.59	−0.60	−0.74	−0.57	−0.79	−0.69	−0.52	−0.74	−0.84
3	0.11	0.16	0.26	0.15	0.35	0.20	0.02	0.26	0.27
4	0.05	0.09	0.01	−0.04	0.04	−0.07	0.13	0.11	0.03
5	0.09	0.11	0.07	0.11	0.08	0.14	0.08	0.05	0.09
6	0.11	0.11	0.09	0.11	0.09	0.10	0.08	0.08	0.06
7	0.02	0.04	0.06	0.03	0.02	0.05	0.03	0.02	0.05
8	0.40	0.39	0.50	0.37	0.51	0.50	0.35	0.48	0.57
9	−0.12	−0.33	−0.13	−0.08	−0.19	−0.30	−0.17	−0.10	−0.23
10	0.07	0.13	0.06	0.06	0.09	0.10	0.09	0.07	0.08
11	0.06	0.08	0.05	0.04	0.06	0.11	0.06	0.03	0.08
12	−0.20	−0.08	−0.06	−0.16	−0.15	0.10	−0.20	−0.18	−0.03
13	0.10	0.11	0.03	0.09	0.12	−0.03	0.08	0.11	0.02
14	0.08	0.03	0.05	0.07	0.05	0.01	0.11	0.06	0.06
15	0.69	0.63	0.66	0.67	0.66	0.65	0.70	0.65	0.63
16	−0.57	−0.50	−0.55	−0.54	−0.51	−0.57	−0.56	−0.50	−0.51
17	−0.58	−0.60	−0.59	−0.60	−0.61	−0.59	−0.61	−0.61	−0.61
18	0.01	0.02	−0.02	0.03	−0.17	0.10	0.11	−0.17	0.07
19	−0.31	−0.40	−0.41	−0.33	−0.37	−0.45	−0.30	−0.37	−0.42
20	0.07	0.10	0.11	0.09	0.09	0.12	0.07	0.09	0.12
21	0.08	0.11	0.10	0.09	0.09	0.12	0.09	0.09	0.10
22	0.09	0.09	0.11	0.09	0.09	0.12	0.07	0.09	0.11
23	−0.75	−0.79	−0.86	−0.74	−0.99	−0.74	−0.74	−0.96	−0.84
24	0.39	0.46	0.45	0.39	0.57	0.33	0.39	0.52	0.38
25	0.16	0.15	0.20	0.15	0.12	0.20	0.15	0.17	0.21
26	−0.50	−0.53	−0.60	−0.47	−0.52	−0.55	−0.48	−0.57	−0.56
27	0.51	0.54	0.66	0.50	0.68	0.56	0.50	0.73	0.64
28	0.78	0.82	0.85	0.76	0.81	0.82	0.77	0.82	0.82
29	−0.59	−0.64	−0.65	−0.57	−0.63	−0.65	−0.56	−0.59	−0.63
30	−0.56	−0.56	−0.56	−0.58	−0.56	−0.55	−0.57	−0.59	−0.56
31	0.15	0.15	0.15	0.16	0.15	0.15	0.15	0.15	0.15
32	0.15	0.15	0.15	0.16	0.15	0.15	0.15	0.15	0.15
33	0.15	0.16	0.15	0.16	0.16	0.15	0.15	0.16	0.16
34	−0.59	−0.62	−0.62	−0.54	−0.67	−0.54	−0.55	−0.65	−0.57
35	0.19	0.19	0.17	0.17	0.19	0.16	0.17	0.18	0.16
36	−0.43	−0.44	−0.46	−0.41	−0.43	−0.43	−0.41	−0.44	−0.44
37	0.13	0.14	0.14	0.13	0.13	0.13	0.12	0.13	0.13
38	0.13	0.14	0.14	0.13	0.13	0.13	0.12	0.12	0.13
39	0.13	0.13	0.13	0.13	0.14	0.12	0.12	0.13	0.13
40	−0.64	−0.55	−0.74	−0.68	−0.75	−0.62	−0.63	−0.76	−0.78
41	0.32	0.28	0.43	0.34	0.38	0.29	0.32	0.43	0.46

	6-fold			9-fold			12-fold		
	α	β	γ	α	β	γ	α	β	γ
42	0.22	0.21	0.16	0.24	0.18	0.32	0.22	0.25	0.18
43	0.04	0.05	0.01	0.05	0.01	−0.02	0.02	−0.02	0.01
44	−0.23	−0.15	−0.20	−0.26	−0.11	−0.27	−0.22	−0.22	−0.21
45	0.07	0.08	0.07	0.07	0.07	0.10	0.06	0.10	0.07
46	0.52	0.41	0.55	0.56	0.51	0.42	0.49	0.57	0.57
47	0.09	0.06	0.19	0.12	0.06	0.15	0.14	0.11	0.19
48	−0.00	0.01	−0.01	−0.02	−0.01	0.01	−0.01	−0.02	0.01
49	0.01	0.01	−0.03	0.01	0.00	−0.02	0.01	0.00	−0.03
50	−0.30	−0.30	−0.34	−0.29	−0.30	−0.30	−0.31	−0.32	−0.33
51	0.08	0.09	0.09	0.06	0.09	0.08	0.06	0.09	0.09
52	0.07	0.08	0.08	0.08	0.07	0.08	0.08	0.09	0.08
53	0.08	0.09	0.08	0.07	0.11	0.08	0.07	0.10	0.08
54	−0.55	−0.55	−0.66	−0.57	−0.59	−0.58	−0.52	−0.65	−0.67
55	0.18	0.19	0.21	0.17	0.18	0.20	0.18	0.21	0.21
56	−0.44	−0.42	−0.34	−0.47	−0.33	−0.36	−0.43	−0.33	−0.34
57	0.11	0.11	0.07	0.12	0.09	0.09	0.11	0.09	0.08
58	0.11	0.10	0.09	0.12	0.08	0.10	0.11	0.09	0.10
59	0.11	0.10	0.10	0.12	0.08	0.08	0.12	0.08	0.09
60	−0.74	−0.80	−0.88	−0.76	−1.02	−0.76	−0.70	−0.95	−0.89
61	0.42	0.50	0.52	0.47	0.66	0.41	0.44	0.56	0.55
62	0.12	0.08	0.07	0.10	0.02	0.12	0.10	0.06	0.06
63	−0.34	−0.30	−0.34	−0.33	−0.31	−0.32	−0.30	−0.33	−0.34
64	0.29	0.30	0.37	0.29	0.48	0.27	0.22	0.47	0.35
65	0.69	0.73	0.72	0.71	0.72	0.68	0.68	0.71	0.79
66	−0.53	−0.55	−0.51	−0.55	−0.55	−0.49	−0.54	−0.55	−0.55
67	−0.64	−0.74	−0.69	−0.71	−0.64	−0.67	−0.67	−0.63	−0.86
68	0.21	0.22	0.24	0.23	0.20	0.22	0.19	0.20	0.25
69	−0.10	−0.18	−0.10	−0.08	−0.10	−0.12	−0.07	−0.11	−0.08
70	0.12	0.13	0.11	0.11	0.09	0.11	0.11	0.13	0.12
71	0.12	0.14	0.10	0.11	0.10	0.12	0.10	0.08	0.09
72	0.10	0.11	0.12	0.10	0.13	0.11	0.09	0.12	0.09
73	0.69	0.73	0.73	0.72	0.74	0.71	0.72	0.78	0.84
74	−0.48	−0.50	−0.49	−0.49	−0.48	−0.46	−0.51	−0.53	−0.52
75	−0.36	−0.34	−0.39	−0.37	−0.37	−0.36	−0.38	−0.39	−0.41
76	−0.62	−0.65	−0.64	−0.68	−0.69	−0.51	−0.63	−0.68	−0.66
77	0.21	0.20	0.19	0.21	0.19	0.18	0.21	0.20	0.19
78	−0.43	−0.44	−0.41	−0.43	−0.43	−0.43	−0.44	−0.44	−0.41
79	0.13	0.14	0.13	0.13	0.14	0.14	0.14	0.14	0.13
80	0.13	0.14	0.13	0.14	0.14	0.13	0.14	0.14	0.13
81	0.13	0.14	0.13	0.14	0.14	0.14	0.14	0.15	0.13

Supporting Figures

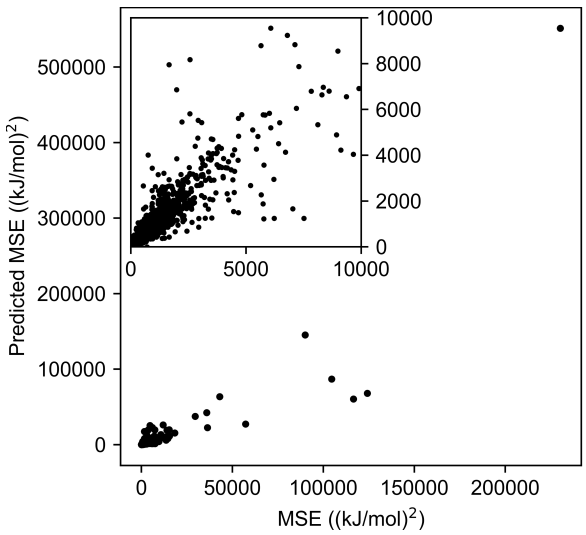

Figure S1: Correlations between the reference and the predicted values of the MSE for the test set.

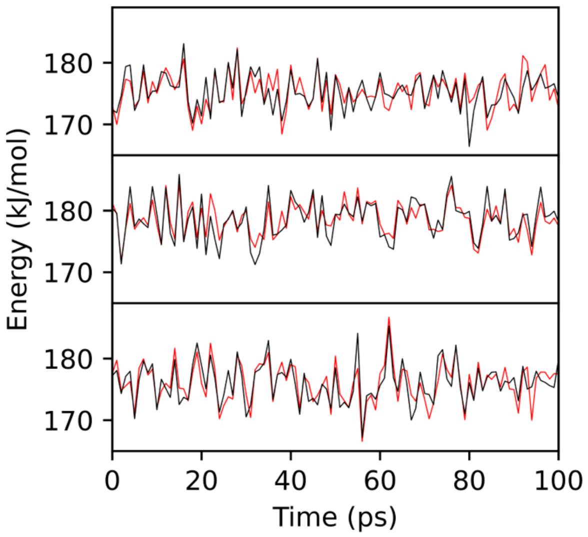

Figure S2: Time profiles of the excitation energies of BChl α, calculated using IM/MM (red) and reference QM/MM results (black) for complexes with 6-fold (top), 9-fold (middle) and 12-fold (bottom) symmetries. Excitation energies from IM/MM are vertically shifted to align with QM/MM results.

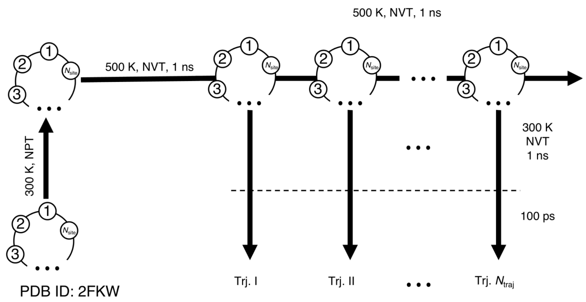

Figure S3: A schematic overview of the sampling protocol. The procedures began with an energy-minimized structure, followed by a 1 ns equilibration in the NPT ensemble. The system was then subjected to a 2 ns annealing process at 500 K using the NVT ensemble. From the final nanosecond, ten snapshots were selected and each was equilibrated at 300 K for 1 ns under NVT conditions. Each trajectory was subsequently extended for an additional 100 ps, which constituted the production run.

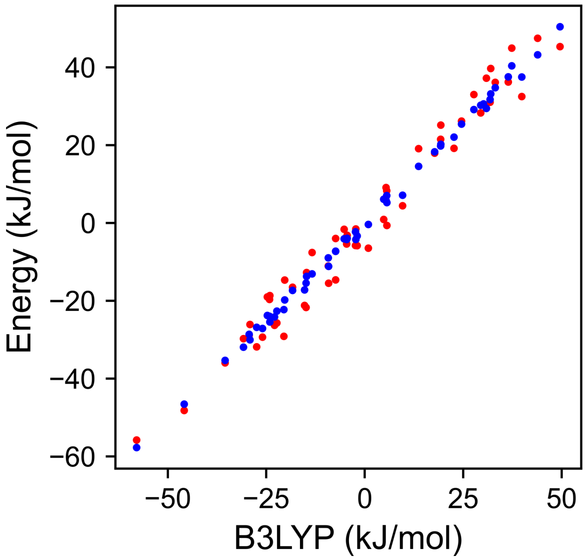

Figure S4: The agreement between B3LYP and two additional DFT methods. The data displays the energies at some trajectory-sampled geometries. The horizontal axis denotes the B3LYP energies, while the vertical axis denotes the energy from the range-separated CAM-B3LYP functional (red) or from the dispersion corrected B3LYP (blue). Because the energies in one method can be shifted by a constant, the energy at an arbitrarily selected geometry was set to zero with all the three DFT methods.

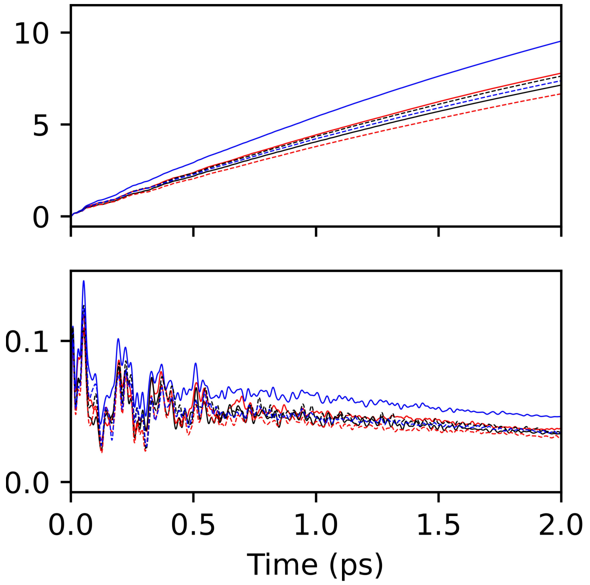

Figure S5: Real (top) and imaginary (bottom) parts of the line shape functions. Solid and dashed lines represent BChl α and β, while red, black, and blue correspond to 6-fold, 9-fold, and 12-fold symmetries.

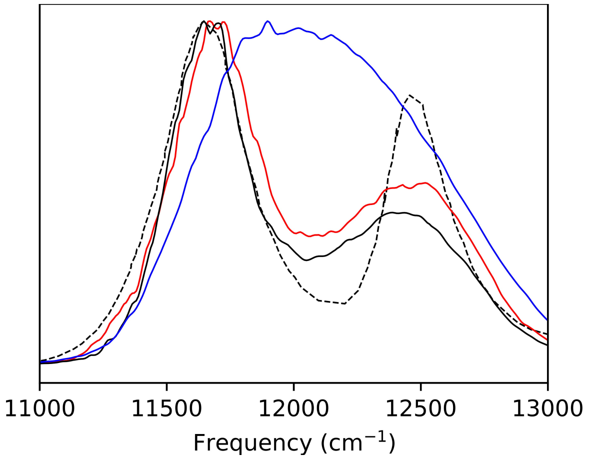

Figure S6: Calculated linear absorption line shape of LH2 complexes with 6-fold (red), 9-fold (black) and 12-fold (blue) symmetries, in comparison with experimental result (dashed). Total standard deviations of excitation energies for the 6-fold (12-fold) complex are 260, 252, 252 cm^{-1} (269, 246, 260 cm^{-1}) for BChl α, β and γ, respectively.

References

(S1) Yanai, T.; Tew, D. P.; Handy, N. C. A new hybrid exchange–correlation functional using the Coulomb-attenuating method (CAM-B3LYP). *Chem. Phys. Lett.* **2004**, *393*, 51–57.

(S2) Grimme, S.; Ehrlich, S.; Goerigk, L. Effect of the damping function in dispersion corrected density functional theory. *J. Comput. Chem.* **2011**, *32*, 1456–1465.

(S3) Jang, S.; Rivera, E.; Montemayor, D. Molecular Level Design Principle behind Optimal Sizes of Photosynthetic LH2 Complex: Taming Disorder through Cooperation of Hydrogen Bonding and Quantum Delocalization. *J. Phys. Chem. Lett.* **2015**, *6*, 928–934.

(S4) Jorgensen, W. L.; Chandrasekhar, J.; Madura, J. D.; Impey, R. W.; Klein, M. L. Comparison of simple potential functions for simulating liquid water. *J. Chem. Phys.* **1983**, *79*, 926–935.

(S5) Pronk, S.; Páll, S.; Schulz, R.; Larsson, P.; Bjelkmar, P.; Apostolov, R.; Shirts, M. R.; Smith, J. C.; Kasson, P. M.; van der Spoel, D.; Hess, B.; Lindahl, E. GROMACS 4.5: a high-throughput and highly parallel open source molecular simulation toolkit. *Bioinformatics* **2013**, *29*, 845–854.

(S6) Epifanovsky, E.; Gilbert, A. T. B.; Feng, X.; Lee, J.; Mao, Y.; Mardirossian, N.; Pokhilko, P.; White, A. F.; Coons, M. P.; Dempwolff, A. L.; Gan, Z.; Hait, D.; Horn, P. R.; Jacobson, L. D.; Kaliman, I.; Kussmann, J.; Lange, A. W.; Lao, K. U.; Levine, D. S.; Liu, J.; McKenzie, S. C.; Morrison, A. F.; Nanda, K. D.; Plasser, F.; Rehn, D. R.; Vidal, M. L.; You, Z.-Q.; Zhu, Y.; Alam, B.; Albrecht, B. J.; Aldossary, A.; Alguire, E.; Andersen, J. H.; Athavale, V.; Barton, D.; Begam, K.; Behn, A.; Bellonzi, N.; Bernard, Y. A.; Berquist, E. J.; Burton, H. G. A.; Carreras, A.; Carter-Fenk, K.; Chakraborty, R.; Chien, A. D.; Closser, K. D.; Cofer-Shabica, V.;

Dasgupta, S.; de Wergifosse, M.; Deng, J.; Diedenhofen, M.; Do, H.; Ehlert, S.; Fang, P.-T.; Fatehi, S.; Feng, Q.; Friedhoff, T.; Gayvert, J.; Ge, Q.; Gidofalvi, G.; Goldey, M.; Gomes, J.; González-Espinoza, C. E.; Gulania, S.; Gunina, A. O.; Hanson-Heine, M. W. D.; Harbach, P. H. P.; Hauser, A.; Herbst, M. F.; Hernández Vera, M.; Hodecker, M.; Holden, Z. C.; Houck, S.; Huang, X.; Hui, K.; Huynh, B. C.; Ivanov, M.; Jász, ; Ji, H.; Jiang, H.; Kaduk, B.; Kähler, S.; Khistyaev, K.; Kim, J.; Kis, G.; Klunzinger, P.; Koczor-Benda, Z.; Koh, J. H.; Kosenkov, D.; Koulias, L.; Kowalczyk, T.; Krauter, C. M.; Kue, K.; Kunitsa, A.; Kus, T.; Ladjánszki, I.; Landau, A.; Lawler, K. V.; Lefrancois, D.; Lehtola, S.; Li, R. R.; Li, Y.-P.; Liang, J.; Liebenthal, M.; Lin, H.-H.; Lin, Y.-S.; Liu, F.; Liu, K.-Y.; Loipersberger, M.; Luenser, A.; Manjanath, A.; Manohar, P.; Mansoor, E.; Manzer, S. F.; Mao, S.-P.; Marenich, A. V.; Markovich, T.; Mason, S.; Maurer, S. A.; McLaughlin, P. F.; Menger, M. F. S. J.; Mewes, J.-M.; Mewes, S. A.; Morgante, P.; Mullinax, J. W.; Oosterbaan, K. J.; Paran, G.; Paul, A. C.; Paul, S. K.; Pavošević, F.; Pei, Z.; Prager, S.; Proynov, E. I.; Rák, ; Ramos-Cordoba, E.; Rana, B.; Rask, A. E.; Rettig, A.; Richard, R. M.; Rob, F.; Rossomme, E.; Scheele, T.; Scheurer, M.; Schneider, M.; Sergueev, N.; Sharada, S. M.; Skomorowski, W.; Small, D. W.; Stein, C. J.; Su, Y.-C.; Sundstrom, E. J.; Tao, Z.; Thirman, J.; Tornai, G. J.; Tsuchimochi, T.; Tubman, N. M.; Veccham, S. P.; Vydrov, O.; Wenzel, J.; Witte, J.; Yamada, A.; Yao, K.; Yeganeh, S.; Yost, S. R.; Zech, A.; Zhang, I. Y.; Zhang, X.; Zhang, Y.; Zuev, D.; Aspuru-Guzik, A.; Bell, A. T.; Besley, N. A.; Bravaya, K. B.; Brooks, B. R.; Casanova, D.; Chai, J.-D.; Coriani, S.; Cramer, C. J.; Cserey, G.; DePrince, I., A. Eugene; DiStasio, J., Robert A.; Dreuw, A.; Dunietz, B. D.; Furlani, T. R.; Goddard, I., William A.; Hammes-Schiffer, S.; Head-Gordon, T.; Hehre, W. J.; Hsu, C.-P.; Jagau, T.-C.; Jung, Y.; Klamt, A.; Kong, J.; Lambrecht, D. S.; Liang, W.; Mayhall, N. J.; McCurdy, C. W.; Neaton, J. B.; Ochsenfeld, C.; Parkhill, J. A.; Peverati, R.; Rassolov, V. A.; Shao, Y.; Slipchenko, L. V.; Stauch, T.; Steele, R. P.; Subotnik, J. E.; Thom, A. J. W.; Tkatchenko, A.; Truh-

lar, D. G.; Van Voorhis, T.; Wesolowski, T. A.; Whaley, K. B.; Woodcock, I., H. Lee; Zimmerman, P. M.; Faraji, S.; Gill, P. M. W.; Head-Gordon, M.; Herbert, J. M.; Krylov, A. I. Software for the frontiers of quantum chemistry: An overview of developments in the Q-Chem 5 package. *J. Chem. Phys.* **2021**, *155*, 084801.

(S7) Kim, C. W.; Rhee, Y. M. Constructing an Interpolated Potential Energy Surface of a Large Molecule: A Case Study with Bacteriochlorophyll a Model in the Fenna–Matthews–Olson Complex. *J. Chem. Theory Comput.* **2016**, *12*, 5235–5246.

(S8) Song, C.-I.; Rhee, Y. M. Development of force field parameters for oxyluciferin on its electronic ground and excited states. *Int. J. Quantum Chem.* **2011**, *111*, 4091–4105.

(S9) MacKerell, A. D. J.; Bashford, D.; Bellott, M.; Dunbrack, R. L. J.; Evanseck, J. D.; Field, M. J.; Fischer, S.; Gao, J.; Guo, H.; Ha, S.; Joseph-McCarthy, D.; Kuchnir, L.; Kuczera, K.; Lau, F. T. K.; Mattos, C.; Michnick, S.; Ngo, T.; Nguyen, D. T.; Prodhom, B.; Reiher, W. E.; Roux, B.; Schlenkrich, M.; Smith, J. C.; Stote, R.; Straub, J.; Watanabe, M.; Wiórkiewicz-Kuczera, J.; Yin, D.; Karplus, M. All-Atom Empirical Potential for Molecular Modeling and Dynamics Studies of Proteins. *J. Phys. Chem. B* **1998**, *102*, 3586–3616.

(S10) Cho, K. H.; Chung, S.; Rhee, Y. M. Efficiently Transplanting Potential Energy Interpolation Database between Two Systems: Bacteriochlorophyll Case with FMO and LH2 Complexes. *J. Chem. Inf. Model.* **2019**, *59*, 4228–4238.

(S11) Bettens, R. P. A.; Collins, M. A. Learning to interpolate molecular potential energy surfaces with confidence: A Bayesian approach. *J. Chem. Phys.* **1999**, *111*, 816–826.
